# Correction: Filter bank common spatial pattern and envelope-based features in multimodal EEG-fTCD brain-computer interfaces

**DOI:** 10.1371/journal.pone.0334075

**Published:** 2025-10-09

**Authors:** The PLOS One Staff

## Notice of Republication

This article was republished on 23^rd^ September 2025, to correct an error in PDF. There was an error in the author contributions that was introduced during type setting. The publisher apologises for this error.

## Supporting information

S1 File Originally published, uncorrected article. (PDF)

S2 File Republished, corrected article. (PDF)
