# Supplementary material for: Filter bank common spatial pattern and envelope-based features in multimodal EEG-fTCD brain-computer interfaces
Source: PLoS One. 2025 May 22;20(5):e0311075. doi: 10.1371/journal.pone.0311075 (PMC12097611; doi:10.1371/journal.pone.0311075)
Supplement: S1 Table — (DOCX) [file pone.0311075.s001.docx]

**S1 Table.** Maximum accuracy achieved for each subject using SVM and the corresponding accuracies obtained using Concatenation and fusion for MI paradigm.

|  | Sub_ID | 1 | 2 | 3 | 4 | 5 | 6 | 7 | 8 | 9 | 10 | **Mean ±STD** |
| --- | --- | --- | --- | --- | --- | --- | --- | --- | --- | --- | --- | --- |
| Baseline vs Left | Concatenation | 98.97 | 96.91 | 81.44 | 86.6 | 93.81 | 90.72 | 79.38 | 95.88 | 92.78 | 82.47 | 89.9± 6.99 |
|  | Fusion | 97.92 | 96.88 | 95.83 | 94.79 | 96.88 | 95.83 | 88.54 | 95.83 | 95.83 | 90.62 | 94.9±2.81 |
|  | | | | | | | | | | | | |
| Baseline vs Right | Concatenation | 98.96 | 89.58 | 86.46 | 84.38 | 95.83 | 91.67 | 81.25 | 87.5 | 92.71 | 83.33 | 89.17±5.67 |
|  | Fusion | 97.89 | 94.74 | 91.58 | 92.63 | 96.84 | 96.84 | 89.47 | 97.89 | 95.79 | 91.58 | 94.53± 2.86 |
|  |  |  |  |  |  |  |  |  |  |  |  |  |
| Right vs Left | Concatenation | 96.19 | 82.86 | 75.24 | 94.29 | 97.14 | 90.48 | 92.38 | 92.38 | 97.14 | 95.24 | 91.33± 7.06 |
|  | Fusion | 99.05 | 85.71 | 89.52 | 98.1 | 100 | 99.05 | 94.29 | 97.14 | 100 | 100 | 96.29± 4.72 |
|  |  |  |  |  |  |  |  |  |  |  |  |  |
