# Supplementary material for: Filter bank common spatial pattern and envelope-based features in multimodal EEG-fTCD brain-computer interfaces
Source: PLoS One. 2025 May 22;20(5):e0311075. doi: 10.1371/journal.pone.0311075 (PMC12097611; doi:10.1371/journal.pone.0311075)
Supplement: S2 Table — (DOCX) [file pone.0311075.s002.docx]

**S2 Table.** Maximum accuracy achieved for each subject using LDA and the corresponding accuracies obtained using Concatenation and fusion for MI paradigm.

|  | Sub_ID | 1 | 2 | 3 | 4 | 5 | 6 | 7 | 8 | 9 | 10 | **Mean ±STD** |
| --- | --- | --- | --- | --- | --- | --- | --- | --- | --- | --- | --- | --- |
| Baseline vs Left | Concatenation | 95.88 | 96.91 | 84.54 | 84.54 | 92.78 | 91.75 | 79.38 | 90.72 | 89.69 | 79.38 | 88.71± 5.98 |
|  | Fusion | 98.97 | 92.78 | 94.85 | 95.88 | 96.91 | 97.94 | 86.6 | 88.66 | 92.78 | 88.66 | 93.4±4.05 |
|  | | | | | | | | | | | | |
| Baseline vs Right | Concatenation | 97.92 | 89.58 | 79.17 | 84.38 | 92.71 | 89.58 | 77.08 | 84.38 | 86.46 | 80.21 | 86.15±6.15 |
|  | Fusion | 97.92 | 88.54 | 94.79 | 82.29 | 98.96 | 93.75 | 81.25 | 92.71 | 91.67 | 88.54 | 91.04± 5.65 |
|  |  |  |  |  |  |  |  |  |  |  |  |  |
| Right vs Left | Concatenation | 94.29 | 79.05 | 78.09 | 81.91 | 91.43 | 87.62 | 82.86 | 85.71 | 87.62 | 85.71 | 85.43± 4.88 |
|  | Fusion | 98.1 | 80 | 89.52 | 93.33 | 95.24 | 92.38 | 93.33 | 96.19 | 98.1 | 97.14 | 93.33± 5.15 |
|  |  |  |  |  |  |  |  |  |  |  |  |  |
