# Supplementary material for: Filter bank common spatial pattern and envelope-based features in multimodal EEG-fTCD brain-computer interfaces
Source: PLoS One. 2025 May 22;20(5):e0311075. doi: 10.1371/journal.pone.0311075 (PMC12097611; doi:10.1371/journal.pone.0311075)
Supplement: S5 Table — (DOCX) [file pone.0311075.s005.docx]

**S5 Table.** Maximum accuracy achieved for each subject using SVM and the corresponding accuracies obtained using Concatenation and fusion for MR/WG paradigm.

|  | Sub_ID | 1 | 2 | 3 | 4 | 5 | 6 | 7 | 8 | 9 | 10 | 11 | **Mean± STD** |
| --- | --- | --- | --- | --- | --- | --- | --- | --- | --- | --- | --- | --- | --- |
| Baseline vs MR | Concatenation | 87.5 | 92.71 | 85.42 | 87.5 | 88.54 | 93.75 | 98.96 | 98.96 | 98.96 | 94.79 | 92.71 | 92.71±4.74 |
|  | Fusion | 93.75 | 96.88 | 89.58 | 91.67 | 96.88 | 95.83 | 96.88 | 96.88 | 98.96 | 95.83 | 94.79 | 95.27± 2.58 |
| Baseline vs WG | Concatenation | 86.6 | 74.23 | 86.6 | 70.1 | 82.47 | 87.63 | 84.54 | 91.75 | 91.75 | 69.07 | 76.29 | 82.47± 7.81 |
|  | Fusion | 91.58 | 92.63 | 89.47 | 72.63 | 85.26 | 86.32 | 87.37 | 93.68 | 95.79 | 67.37 | 83.16 | 85.93± 8.42 |
|  |  |  |  |  |  |  |  |  |  |  |  |  |  |
| MR vs WG | Concatenation | 93.33 | 99.05 | 92.38 | 84.76 | 93.33 | 99.05 | 90.48 | 98.09 | 100 | 97.14 | 99.05 | 94.76± 4.53 |
|  | Fusion | 97.14 | 99.05 | 98.1 | 86.67 | 97.14 | 100 | 95.24 | 98.1 | 99.05 | 98.1 | 98.1 | 96.97± 3.48 |
