# Supplementary material for: Filter bank common spatial pattern and envelope-based features in multimodal EEG-fTCD brain-computer interfaces
Source: PLoS One. 2025 May 22;20(5):e0311075. doi: 10.1371/journal.pone.0311075 (PMC12097611; doi:10.1371/journal.pone.0311075)
Supplement: S6 Table — (DOCX) [file pone.0311075.s006.docx]

**S6 Table.** Maximum accuracy achieved for each subject using LDA and the corresponding accuracies obtained using Concatenation and fusion for MR/WG paradigm.

|  | Sub_ID | 1 | 2 | 3 | 4 | 5 | 6 | 7 | 8 | 9 | 10 | 11 | **Mean± STD** |
| --- | --- | --- | --- | --- | --- | --- | --- | --- | --- | --- | --- | --- | --- |
| Baseline vs MR | Concatenation | 85.42 | 88.54 | 86.46 | 88.54 | 84.38 | 84.38 | 95.83 | 97.92 | 100 | 92.71 | 89.58 | 90.34±5.26 |
|  | Fusion | 91.67 | 94.79 | 90.62 | 91.67 | 89.58 | 91.67 | 96.88 | 97.92 | 98.96 | 97.92 | 84.38 | 93.28± 4.25 |
| Baseline vs WG | Concatenation | 83.5 | 68.04 | 82.47 | 65.98 | 73.2 | 85.57 | 74.23 | 91.75 | 90.72 | 64.95 | 72.17 | 77.51± 9.22 |
|  | Fusion | 88.66 | 95.88 | 84.54 | 72.16 | 74.23 | 82.47 | 84.54 | 95.88 | 93.81 | 69.07 | 80.41 | 83.79± 8.91 |
|  |  |  |  |  |  |  |  |  |  |  |  |  |  |
| MR vs WG | Concatenation | 86.67 | 97.14 | 93.33 | 90.48 | 84.76 | 96.19 | 88.57 | 96.19 | 100 | 96.19 | 96.19 | 93.25± 4.67 |
|  | Fusion | 92.38 | 100 | 97.14 | 89.52 | 97.14 | 97.14 | 93.33 | 98.1 | 100 | 99.05 | 96.19 | 96.36± 3.17 |
