# Supplementary material for: Filter bank common spatial pattern and envelope-based features in multimodal EEG-fTCD brain-computer interfaces
Source: PLoS One. 2025 May 22;20(5):e0311075. doi: 10.1371/journal.pone.0311075 (PMC12097611; doi:10.1371/journal.pone.0311075)
Supplement: S8 Table — (DOCX) [file pone.0311075.s008.docx]

**S8 Table.** P-values showing accuracy significance of fusion compared to Concatenation for the MR/WG paradigm with LDA.

| Comparison | Baseline vs MR | Baseline vs WG | MR vs WG |
| --- | --- | --- | --- |
| Fusion/Concatenation | 0.0160 | 0.0034 | 0.0075 |
