# Supplementary material for: Correction: Filter bank common spatial pattern and envelope-based features in multimodal EEG-fTCD brain-computer interfaces
Source: PLoS One. 2025 Oct 9;20(10):e0334075. doi: 10.1371/journal.pone.0334075 (PMC12510475; doi:10.1371/journal.pone.0334075)
Supplement: S2 File — (PDF) [file pone.0334075.s002.pdf]

RESEARCH ARTICLE

Filter bank common spatial pattern and envelope-based features in multimodal EEG-fTCD brain-computer interfaces

Alaa-Allah Essam[1☯], Ammar Ibrahim[1☯], Ashar Seif Al-Nasr[1☯], Mariam El-Saqa[1☯], Sohila Mohamed[1☯], Ayman Anwar[1,2], Ayman Eldeib[1,3], Murat Akcakaya[4], Aya Khalaf[1,5*]

1 Biomedical Engineering and Systems Department, Faculty of Engineering, Cairo University, Giza, Egypt, **2** Department of Electrical and Computer Engineering, University of Toronto, Ontario, Canada, **3** Computer Science Department, School of Engineering, Technology, and Aeronautics (SETA), Southern New Hampshire University (SNHU), New Hampshire, United States of America, **4** Electrical and Computer Engineering Department, University of Pittsburgh, Pittsburgh, Pennsylvania, United States of America, **5** Department of Neurology, Yale University School of Medicine, New Haven, Connecticut, United States of America

☯ Equally contributing co-first authors.
* aya.khalaf@yale.edu

OPEN ACCESS

Citation: Essam A-A, Ibrahim A, Seif Al-Nasr A, El-Saqa M, Mohamed S, Anwar A, et al. (2025) Filter bank common spatial pattern and envelope-based features in multimodal EEG-fTCD brain-computer interfaces. PLoS One 20(5): e0311075. https://doi.org/10.1371/journal.pone.0311075

Editor: Umer Asgher, NUST: National University of Sciences and Technology, PAKISTAN

Received: September 12, 2024

Accepted: March 10, 2025

Published: May 22, 2025

Copyright: © 2025 Essam et al. This is an open access article distributed under the terms of the Creative Commons Attribution License, which permits unrestricted use, distribution, and reproduction in any medium, provided the original author and source are credited.

Data availability statement: Datasets included in the study are available through Dryad at https://doi.org/10.5061/dryad.k0p2ngfk1.

Funding: The author(s) received no specific funding for this work.

Abstract

Brain-computer interfaces (BCIs) provide alternative means of communication and control for individuals with severe motor or speech impairments. Multimodal BCIs have been introduced recently to enhance the performance of BCIs utilizing single modality. In this paper, we aim to advance the state of the art in multimodal BCIs combining Electroencephalography (EEG) and functional transcranial Doppler ultrasound (fTCD) by introducing advanced analysis approaches that enhance system performance. Our EEG-fTCD BCIs employ two distinct paradigms to infer user intent: motor imagery (MI) and flickering mental rotation (MR)/word generation (WG) paradigms. In the MI paradigm, we introduce the use of Filter Bank Common Spatial Pattern (FBCSP) for the first time in an EEG-fTCD BCI, while in the flickering MR/WG paradigm, we extend FBCSP application to non-motor imagery tasks. Additionally, we extract previously unexplored time-series features from the envelope of fTCD signals, leveraging richer information from cerebral blood flow dynamics. Furthermore, we employ a Bayesian fusion framework that allows EEG and fTCD to contribute unequally to decision-making. The multimodal EEG-fTCD system achieved high classification accuracies across tasks in both paradigms. In the MI paradigm, accuracies of 94.53%, 94.9%, and 96.29% were achieved for left arm MI vs. baseline, right arm MI vs. baseline, and right arm MI vs. left arm MI, respectively – outperforming EEG-only accuracy by 3.87%, 3.80%, and 5.81%, respectively. In the MR/WG paradigm, the system achieved 95.27%, 85.93%, and 96.97% for MR vs. baseline, WG vs. baseline, and MR vs. WG, respectively, showing accuracy improvements of 2.28%, 4.95%, and 1.56%, respectively compared to EEG-only results. Overall, the proposed

Competing interests: The authors have declared that no competing interests exist.

analysis approach improved classification accuracy for 5 out of 6 binary classification problems within the MI and MR/WG paradigms, with gains ranging from 0.64% to 9% compared to our previous EEG-fTCD studies. Additionally, our results demonstrate that EEG-fTCD BCIs with the proposed analysis techniques outperform multimodal EEG-fNIRS BCIs in both accuracy and speed, improving classification performance by 2.7% to 24.7% and reducing trial durations by 2–38 seconds. These findings highlight the potential of the proposed approach to advance assistive technologies and improve patient quality of life.

1. Introduction

Brain computer interface (BCI) is a technology aiming at providing a direct communication channel between the central nervous system and external devices [1,2]. Therefore, BCIs can assist individuals suffering from disorders that limit their ability of interaction with the surrounding environment such as stroke, amyotrophic lateral sclerosis, cerebral palsy or spinal cord injury by providing alternative means of communication [3]. Other BCI applications include controlling robots [4], prosthetic limbs [5], rehabilitation [6], virtual reality [7] and neurogaming [8].

Both invasive and non-invasive neuroimaging modalities have been used to develop BCIs. Non-invasive BCIs are safer, offering high accessibility, cost effectiveness, and scalability [9,10]. Different noninvasive neuroimaging modalities capturing the electrical and metabolic activity of the brain have been used in BCI design including electroencephalography (EEG) [11,12], functional near-infrared spectroscopy (fNIRS) [13], functional magnetic resonance imaging (fMRI) [14] and magnetoencephalography (MEG) [15]. Among these modalities, EEG is the most commonly used modality due to its portability, cost-effectiveness and high temporal resolution [16]. However, it has a low signal to noise ratio and low spatial resolution. In addition, it is prone to non-stationarities resulting from external electrical interference and internal brain background activity [17]. These drawbacks limit the performance of EEG-based BCIs outside laboratory-controlled environments and lead to misidentification of user intent and false-command generation [18].

To improve the performance of EEG-based BCIs, various approaches have been proposed, including enhancements in feature extraction techniques and the development of hybrid BCI designs. Recent work focused on improving classification accuracy and enhancing robustness by integrating different evoked potentials [19,20,21]. Other studies introduced signal detection approaches for steady-state visually evoked potentials (SSVEPs) to improve reliability in real-world applications [22,23]. Traditional feature extraction approaches, such as Common Spatial Patterns (CSP), have been widely used to separate EEG motor imagery tasks [24]. Filter bank CSP (FBCSP) was introduced to enhance feature extraction across multiple frequency bands [25]. To improve robustness of CSP, L1-Norm-based feature selection and Dempster-Shafer theory-based optimization were proposed [26]. Different classifiers such as Support Vector Machines (SVM), Linear Discriminant Analysis

(LDA), and Multi-Layer Perceptron (MLP) were explored to infer user intent [27,28,29]. Recently, deep learning methods were introduced to extract multi-scale spatial, temporal, and frequency-domain features, such as Frequency Deformable Convolutional Networks (FDCN-C) [30], Multi-Scale Convolutional Neural Networks (MS-CNN) [31], Adaptive Transfer Learning-based CNN (MSFFCNN) [32], Multiscale Spatial-Temporal Feature Fusion Neural Network (MSTFNet) [33] and Multi-Scale Deep Convolutional Neural Networks (MOCNN) [34]. While these models have demonstrated strong classification performance, their significant computational requirements and extended training times remain significant challenges, limiting their practicality for real-time BCI deployment

Multimodal BCIs employing EEG in addition to other modalities measuring different brain activities such as fNIRS and fMRI were proposed to overcome the limitations of single-modality EEG-based BCIs [35]. However, EEG-fMRI BCIs cannot be implemented in practice due to their non-portability, high cost, and the need for a highly controlled environment for efficient performance [36,37]. fNIRS is the most commonly used second modality in multimodal BCI systems due to its portability and immunity against electrical noise, however, it suffers from low temporal resolution [38–40]. Additionally, infrared signals can be blocked by the user's hair [41,42]. These limitations hinder the applicability of EEG-fNIRS multimodal BCIs in real-time applications.

Functional transcranial Doppler ultrasound (fTCD) has been suggested as a fast and cost-effective alternative for fNIRS in BCI design, offering faster response times and simpler setup complexity [43]. fTCD assesses the cerebral blood flow velocity (CBFV) using two ultrasound transducers placed above the zygomatic arch on both left-side and right-side transtemporal windows [44]. Recently, we proposed a multimodal BCI combining EEG and fTCD modalities as a faster and more efficient alternative to EEG-fNIRS BCIs [45–49]. This BCI employed two different paradigms to induce simultaneous changes in EEG and fTCD through presenting visual stimuli that instruct participants to perform motor imagery tasks (MI paradigm) [46,47] as well as flickering mental rotation (MR) and word generation (WG) tasks (flickering MR/WG paradigm) [45,48].

For fTCD, most studies employed wavelet decomposition, which effectively captures temporal and frequency-domain features but is computationally intensive and less applicable for real-time systems. Our study instead uses time-series features from the fTCD envelope, preserving critical information while significantly reducing computational demands. This approach improved classification accuracy by 9% in mental rotation tasks compared to state-of-the-art EEG-fTCD systems. Significant fTCD features were selected using the Wilcoxon rank-sum test, and classification was performed using a linear kernel SVM.

In this paper, we aim at improving the performance of MI and MR/WG EEG-fTCD BCIs through applying analysis approaches that have not been previously used to analyze multimodal EEG-fTCD data. Specifically, we apply Filter Bank Common Spatial Pattern (FBCSP) for the first time in an MI-based EEG-fTCD BCI and expand its use to non-motor imagery tasks in the flickering MR/WG EEG-fTCD BCI. Furthermore, we extract novel time-series features from the envelope of fTCD signals. Moreover, we investigate the contribution of each modality in user intent inference within each paradigm. Three binary classification problems were investigated for each paradigm, including left MI versus baseline, right MI versus baseline, and left MI versus right MI for the MI paradigm as well as MR versus baseline, WG versus baseline, and WG versus MR for the flickering MR/WG paradigm.Rather than concatenating feature vectors from EEG and fTCD, we implemented a probabilistic Bayesian fusion approach, which assumes that EEG and fTCD provide independent but unequally weighted evidence.

2. Materials and methods

2.1. Data acquisition and preprocessing

EEG data were collected using a g.tec system with 16 electrodes placed according to the 10–20 system at positions Fp1, Fp2, F3, F4, Fz, Fc1, Fc2, Cz, P1, P2, C1, C2, Cp3, Cp4, P5, and P6, with the reference electrode placed at the left mastoid. The acquired signals were sampled at 256 samples/sec. fTCD data were collected at a sampling rate of

44.1 kHz using a SONARA TCD system with two 2 MHz transducers placed at the left and right sides of the transtemporal window, located above the zygomatic arch. A total of 21 healthy participants (age range: 23–32 years) participated in the study, completing a single 25-minute session. The flickering MR/WG paradigm dataset includes data from 11 right-handed participants (3 females), and the MI paradigm dataset includes data from 10 right-handed participants (6 females). Written informed consent was obtained from all participants, and the study was approved by the University of Pittsburgh's Institutional Review Board (IRB) under IRB number PRO16080475. Data collection occurred between April 17 and September 22, 2017.

The EEG data were bandpass filtered during acquisition using the g.tec amplifier's filters, with corner frequencies at 2 and 62 Hz and a notch filter applied between 58 and 62 Hz to remove power line noise. This filtering step ensured that the EEG data were within the desired frequency range, removing unwanted artifacts before analysis. For the fTCD data, downsampling was performed to reduce computational demands. Specifically, the data were downsampled by a factor of 5 using a low-pass filter with a corner frequency of 4.4 kHz, resulting in a final sampling rate of 8.82 kHz.

2.2. Experimental design

The multimodal BCI system employs two visual presentation paradigms to induce simultaneous changes in EEG and fTCD recorded signals. The first paradigm uses motor imagery (MI) tasks while the second one uses flickering mental rotation (MR) and word generation (WG) tasks. Fig 1A shows the MI paradigm visual presentation [47] with three icons presented on the screen including left and right horizontal arrows representing left arm MI and right arm MI tasks respectively, and a fixation cross representing resting state. When the left arm MI task is selected by the vertical red arrow, participants imagine moving their left arm. Similarly, right arm movement is imagined if the right arm MI task is selected. During the experiment, the vertical arrow points randomly to one of the three icons for 10 s (trial duration) and participants perform the task specified by the vertical arrow. In our system, the rest task – where the user focuses on the fixation cross – is randomly selected by the vertical arrow and treated as a separate task, rather than enforcing a rest period after each trial. This design simulates a scenario where the user chooses not to issue any commands. A total of 150 trials were presented to each participant. Fig 1B shows the flickering MR/WG paradigm visual presentation [45] with three icons presented on the screen. The left icon is a random letter representing the WG task. When selected by the vertical red arrow, participants think of words that begin with the letter displayed on the screen. The right icon shows identical 3D shapes rotated with different angles representing the MR task. When selected by the vertical arrow, participants mentally rotate the shapes to decide if they are identical or mirrored. Finally, the icon in the middle is a fixation cross representing resting state. MR and WG tasks can be distinguished through fTCD due to the differences in blood perfusion they yield on the two sides of the brain [43]. Because these tasks do not induce differences in EEG signal, MR/WG tasks were modified to induce differences in EEG signal by being textured with a flickering checkerboard pattern as shown in Fig 1B. This flickering pattern induces a steady-state visually evoked potential (SSVEPs) in EEG. The MR task was modified to flicker at 7 Hz while the WG task was modified to flicker at 17 Hz. Similar to the MI paradigm, the vertical arrow points randomly to one of the three icons for 10 s (trial duration) with a total of 150 trials presented to each participant. Fig 1C shows the multimodal system setup during one of the data collection sessions while Fig 1D shows a schematic illustrating EEG electrodes and fTCD transducers placement. A flowchart of the sequence of events during each data collection session is shown in Fig 2.

2.3. EEG feature extraction

The Common Spatial Pattern (CSP) algorithm is widely employed in MI EEG-based BCIs for feature extraction [24,50]. CSP identifies spatial filters that linearly transform EEG signals from two classes into a new space, where the variance of one class is maximized while the variance of the other is minimized. This enhances the separability of EEG observations

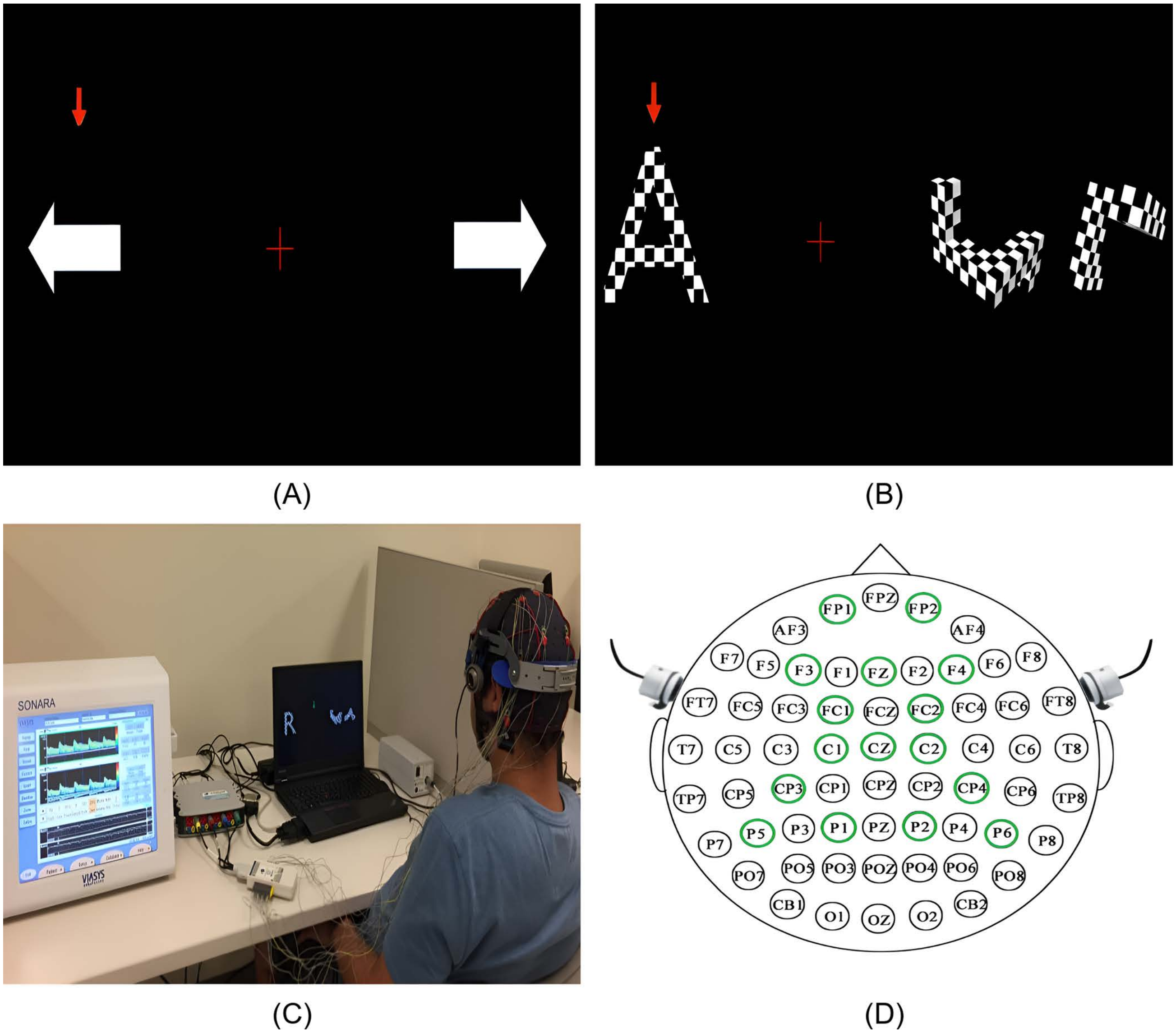

Fig 1. Stimulus presentation for the motor imagery paradigm (A) and the flickering MR/WG paradigm (B), along with (C) the multimodal system setup captured during a data collection session [45] and (D) a schematic illustrating EEG electrodes and fTCD transducers placement.

https://doi.org/10.1371/journal.pone.0311075.g001

associated with distinct tasks. However, CSP performance is highly dependent on subject-specific parameters, such as the selected frequency range, which must be manually specified for each individual [51]. Incorrect or suboptimal frequency selection significantly degrades the performance of the algorithm [52].

To address these limitations, Filter Bank Common Spatial Pattern (FBCSP) was proposed [25]. FBCSP improves upon CSP by automatically dividing the EEG signal into multiple frequency bands and applying CSP to each band separately. This eliminates the need for manual frequency selection and ensures that relevant frequency components (e.g., motor rhythms or steady-state visual evoked potentials) are captured more effectively [51,53]. In this study, FBCSP was applied to EEG data of both paradigms. While FBCSP is well-known as a feature extractor for MI EEG data, in this work, we extend FBCSP applications and show that it can be a successful feature extraction method when applied to SSVEP MR/

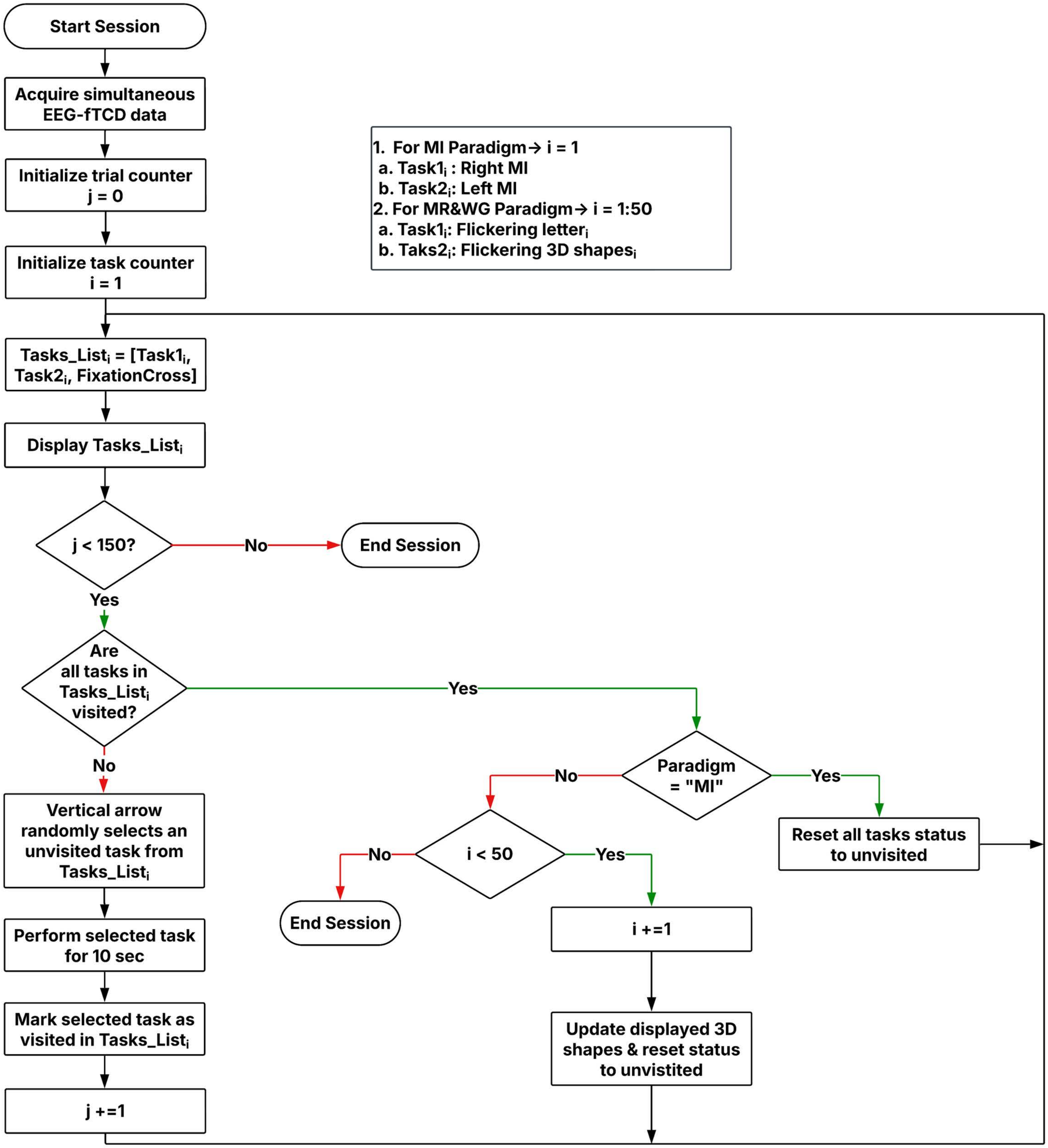

Fig 2. Flowchart illustrating the temporal sequence of events in each data collection session.

https://doi.org/10.1371/journal.pone.0311075.g002

WG and MI EEG data. FBCSP first splits the EEG signals from both paradigms into multiple frequency bands using band-pass filters. In this study, the EEG signals from both paradigms were bandpass filtered in the 2–60 Hz frequency range to ensure the SSVEP related changes in the MR/WG paradigm as well the event-related synchronization and desynchronization rhythms in the MI paradigms are fully represented in the EEG signal [54,55]. The 2–60 Hz frequency range was divided into 9 non-overlapping bands, each having a bandwidth of 6.5 Hz. Within each frequency band, CSP computes spatial filters that maximize the variance differences between the two classes (e.g., task vs. rest). These filters effectively highlight features in the EEG signal that are most relevant for distinguishing between the classes of interest. Within each frequency band, CSP finds optimal spatial filters through solving the equations below [56,57]:

For a given trial, EEG signal can be represented by a matrix E^{N*T} where N is the number of channels and T is the number of samples per channel. Covariance C of each trial is calculated as:

$$C = \frac{E\,E^T}{trace\,(E\,E^T)} \tag{1}$$

The class-specific average covariance matrices are:

$$\Sigma_{c\,\in\,\{+,-\}} = \frac{1}{M}\sum\nolimits_{m=1}^{M} C_m \tag{2}$$

Where M is the number of trials in class c. CSP computes spatial filters W by solving the optimization problem that maximizes the variance difference between two classes:

$$max_w W^T \Sigma_c W$$

$$\begin{aligned} &max_w W^T \Sigma_c W \\ &\text{s.t. } W^T(\Sigma_+ + \Sigma_-)W = 1 \end{aligned} \tag{3}$$

where Σ_c is the average trial covariance matrix for class c ∊ {+, -} and $W^T\Sigma_c W$ is the variance in direction W. Simultaneous diagonalization of $\Sigma_c$matrices can find the optimal spatial filters matrix W from Equation (3).

$$W^T\Sigma_{(+)}\,W = \wedge_{(+)}$$

$$W^T\Sigma_{(-)}\,W = \wedge_{(-)}$$

$$such\ \ that\ \wedge_{(+)} + \wedge_{(-)} = I \tag{4}$$

where $\wedge_c$ is the eigenvalue diagonal matrix. Solution of (4) is equivalent to solution the generalized eigenvalue problem in (5)

$$\Sigma_{(+)}\,w_j = \lambda\,\Sigma_{(-)} w_j \tag{5}$$

Where w_j is the jth generalized eigenvector and $\lambda = \frac{\lambda_j^{(+)}}{\lambda_j^{(-)}}$ represents the class separability for the spatial filter.

$$\lambda_j^{(c)} = w_j{}^T \Sigma_c w_j \tag{6}$$

Where $\lambda_j^{(c)}$ are the diagonal elements of $\wedge_c$. $\lambda_j^{(+)} + \lambda_j^{(-)} = 1$ given that $\wedge(+) + \wedge(-) = I$. Therefore, a higher value of $\lambda_j^{(+)}$ corresponds to a lower value of $\lambda_j^{(-)}$, leading to increased variance in the positive class after filtering using the spatial filter w_j, while simultaneously reducing variance in the negative class after filtering using the same spatial filter w_j.

Since the optimal number of eigenvectors that maximizes the separation the two classes of interest depends on various factors, including the mental tasks being distinguished and the number and placement of EEG electrodes, we tested the classification performance for the binary classification problems of both paradigms at all possible numbers of eigenvectors. In particular, we spatially filtered EEG data using 1, 2, 3, …., and 8 eigenvectors from both ends of W. Therefore, performance of both EEG only and multimodal combination was evaluated using $2N_f$(2, 4, 6, …., and 16) eigenvectors for each frequency band. CSP features included the log variance of each spatially filtered signal. This yielded $2N_f$EEG features per band. Features calculated for EEG bands were concatenated to form the EEG features vector which contained 9x2$N_f$features per trial.

2.4. fTCD Feature Extraction

Studies performing fTCD signals analysis commonly extract features from the raw fTCD data [43,58]. In this study, we extracted features from the fTCD envelope signal, which is derived from the raw fTCD signal captured by the transducers. fTCD envelope signal represents maximal blood flow velocity, while raw fTCD signal represents the echoes recorded by the transducers due to many scatterers moving with different velocities [37]. To convert the amplitudes of the raw fTCD signal to velocities, the Doppler effect equation below was used.

$$f_d = f_r - f_t = \frac{2\, f_t\, v\, coscos\, \theta}{c} \tag{7}$$

Where f_t is the transmitted frequency, f_r is the received frequency, $f_d$is the doppler shift due to the velocity of the scatterers, c is the speed of sound in tissue (1560 cm/s), v is the velocity of the scatterer, and θ is the angle between the ultrasound wave and the flow direction. From this equation, it can be noted that scatterers moving with the highest speed cause the maximal frequency shift. To calculate the envelope signal from raw fTCD data, short-time Fourier transform (STFT) is used to obtain the spectrogram of the raw signal and the maximal frequency which corresponds to the highest blood flow velocity. f_d is plugged into the doppler equation to obtain the corresponding velocity. Fig 3 details the process of calculating the envelope for a sample raw fTCD signal of one trial acquired from the left middle cerebral artery of a single subject.

Time series feature extraction library (TSFEL) was employed to extract features from fTCD envelope signals. Given the lack of established fTCD feature sets in literature, we employed an exploratory approach to extract a comprehensive set of statistical, spectral, and temporal features using TSFEL. This allowed us to capture diverse signal characteristics without restricting the analysis to predefined feature sets. The library has been used to extract statistical features for wavelet coefficients, as these features have been proved to be successful in fTCD-only BCIs [43]. Moreover, we used the library to calculate various sets of features, specifically, statistical features that capture signal distribution and variability (including histogram statistics, variance, and empirical cumulative distribution metrics), spectral features that characterize frequency components through both Fourier and wavelet analysis, and temporal features that quantify signal evolution over time using metrics like autocorrelation and peak characteristics. A complete list of features computed by the algorithm is summarized in Fig 4.

2.5. Feature selection and classification

The Wilcoxon rank-sum test [59] was used to select significant features from fTCD feature vectors of both the MI and MR/WG paradigms at p-value of 0.05. SVM classifier with linear kernel [49] was used to evaluate the performance of single-modal BCIs, i.e., EEG only and fTCD only of both paradigms using a 10-fold cross validation scheme. fTCD only performance was evaluated using features selected at p-value of 0.05 while EEG only performance was evaluated using $2N_f$ $(2,\ 4,\ 6,\ ....,\ and\ 16)$ CSP features per frequency band. fTCD only accuracies as well as best EEG only accuracies were reported in the results section below.

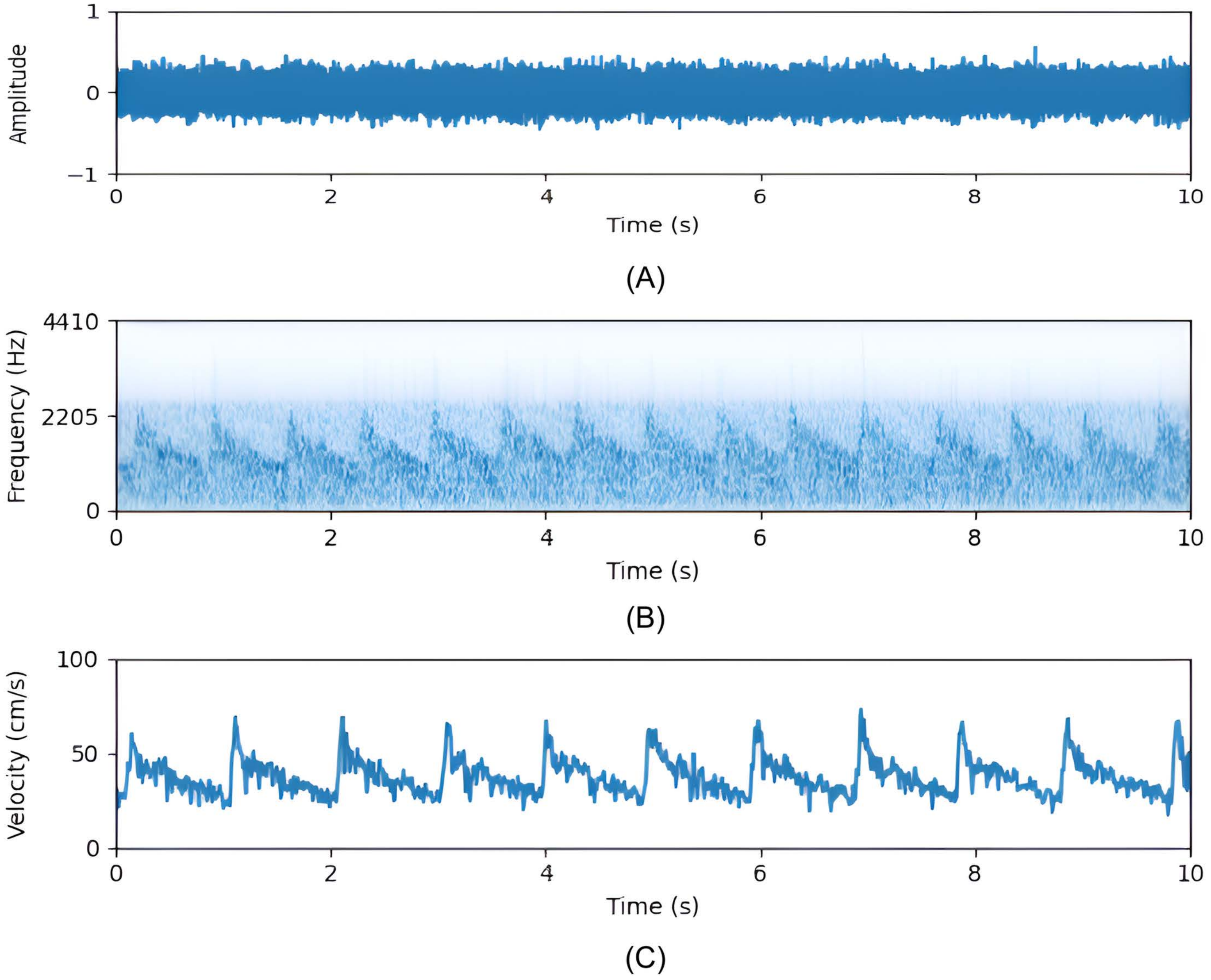

Fig 3. fTCD envelope calculation: (A) raw fTCD signal of a sample trial, (B) spectrogram of raw fTCD signal, and (C) the calculated envelope signal.

https://doi.org/10.1371/journal.pone.0311075.g003

To assess the multimodal BCI performance, SVM was used to project EEG features of each trial into 1-D scalar score (evidence). Similarly, another SVM was used to project fTCD features of each trial into 1-D scalar score (evidence). The fTCD scores were evaluated using fTCD features selected at a p-value of 0.05 while the EEG scores were evaluated using $2N_f$ $(2,\ 4,\ 6,\ ....,$ *and* $16)$CSP features per frequency band. Bayesian fusion was used to infer user intent based on EEG and fTCD scores (evidences). Best multimodal accuracies were reported in the results section below.

2.6. Bayesian Probabilistic Fusion

To generate a joint decision of a test trial based on information from both modalities, we performed a probabilistic Bayesian fusion of EEG and fTCD evidences obtained from the training trials under the assumption that these evidences come from independent distributions, and may have equal or unequal weight in user intent inference [47].

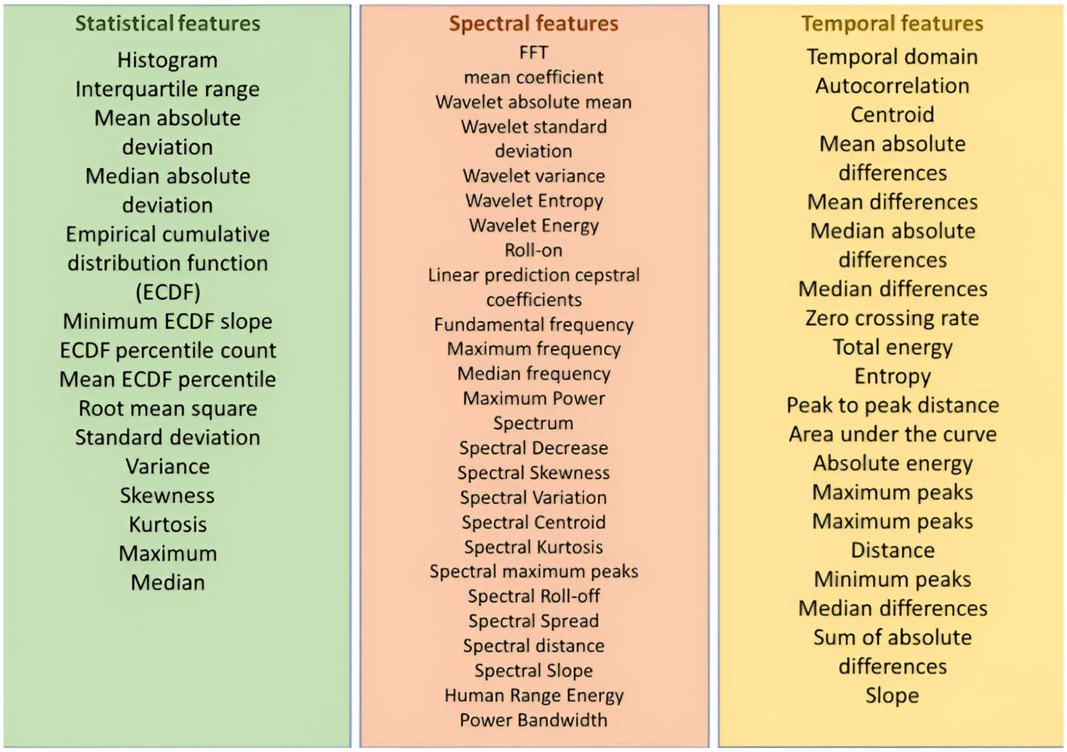

Fig 4. A complete list of features provided by TSFEL.

https://doi.org/10.1371/journal.pone.0311075.g004

2.6.1. Weighted Independent Probabilistic Fusion.

The 1-D SVM scores generated from each modality, also called evidences, are split into training and testing sets using 10-fold cross validation. The goal is to infer the user intent X_k of a test trial given a set of paired evidences $Y = \{y_1, y_2, \ldots y_{N-10}\}$ obtained from the training data where N is the number of trials, and each element of this set $y_n = \{e_n, f_n\}$ represents the EEG (e_n)and fTCD (f_n)evidences of one trial. The user intent $X_k$of a test trial $y_k = \{e_k, f_k\}$ is determined through joint state estimation using EEG and fTCD evidences as follows:

$$\widehat{X}_k = argmax_{X_k}(p(X_k|Y = y_k)) \tag{8}$$

where $p(X_k|Y)$ is the state posterior distribution conditioned on Y. Using Bayes rule, (8) can be formulated as:

$$\widehat{X}_k = argmax_{X_k}(p(Y = y_k|X_k)p\,(X_k)) \tag{9}$$

where $p(Y|X_k)$ is the state conditional distribution of Y and $p\,(X_k)$ is the prior distribution of X_k. Since the trials are randomized, the prior distribution is assumed to be uniform. Consequently, (9) can be written as:

$$\widehat{X}_k = argmax_{X_k}(p(Y = y_k|X_k\,)) \tag{10}$$

The distribution $p(Y|X_k\,))$ can be computed Using EEG and fTCD evidences of the training trials.

Assuming that the EEG and fTCD evidences conditioned on X_k are independent, (10) can be written as follows:

$$\widehat{X}_k = argmax_{X_k}(p(e = e_k|X_k)p(f = f_k|X_k)) \tag{11}$$

The distributions $p(e|X_k)$ and $p(f|X_k)$ represent the EEG and fTCD evidences distributions conditioned on X_k, and are computed in each fold from the N-10 training scores using kernel density estimation with a gaussian kernel and Scott's rule of thumb as the bandwidth selector. The probabilities $p(e = e_k|X_k)$ and $p(f = f_k|X_k)$ for each test trial are computed from the distributions and plugged in equation (11), and the decision $\hat{X}_k$ that maximizes the likelihood is selected. Equation 15 was modified to allow for the possibility that EEG and fTCD evidences does not have equal contribution in decision making, yielding:

$$\widehat{X}_k = argmax_{X_k}(p(e = e_k|X_k)^{\alpha} p(f = f_k|X_k)^{1-\alpha}) \quad (12)$$

where α is a weighting factor determined via a grid search from 0 to 1 with a step of 0.01.

3. Results

In this section, we evaluate the performance of the MI and MR/WG EEG-fTCD systems when employing the proposed analysis pipeline which includes FBCSP for EEG feature extraction and time series features for fTCD envelope feature extraction with weighted Bayesian fusion for multimodal decision making. The following experimental results provide accuracy comparisons of the MI and MR/WG multimodal BCIs against the highest performance achieved using EEG-only and fTCD-only BCIs. Python 3.8 was used to run the experiments on an MSI Cyborg 15 A13VF laptop with a 13th Gen Intel Core i7-13620H CPU (2.4 GHz) and 16 GB RAM.

3.1. Motor imagery paradigm

Table 1 shows maximum accuracy achieved for each subject in right MI versus baseline, left MI versus baseline, and right MI versus left MI selection problems respectively using weighted probabilistic fusion and the corresponding accuracies using EEG only and fTCD only. In order to assess the significance of the multimodal model, one-sided paired Wilcoxon signed rank test was used to statistically compare the accuracies of the multimodal system with the accuracies obtained using EEG only. Table 1 demonstrates that the average accuracies for right MI versus baseline selection problem are 90.73% ± 6.04 for EEG only, 51.98% ± 8.13 for fTCD only, and 94.53% ± 2.86 for the weighted probabilistic fusion. Accuracies obtained using the weighted probabilistic fusion model are statistically significant compared to those obtained using EEG only with a p-value of 0.042 (Table 2). Left MI versus baseline performance measures in Table 1 show average accuracies of 91.03% ± 5.52 and 52.58% ± 7.79 for EEG only and fTCD only respectively while the weighted probabilistic fusion achieved 94.9% ± 2.86. The weighted probabilistic fusion model resulted in a statistically significant increase in accuracy with a p -value of 0.0098 compared to EEG only as shown in Table 2. The third task, right MI versus left MI,

Table 1. Maximum accuracy achieved for each subject using weighted fusion and the corresponding accuracies obtained using EEG only and fTCD only for MI paradigm.

	Sub_ID	1	2	3	4	5	6	7	8	9	10	Mean ±STD
Baseline vs Left	EEG	96.91	95.88	80.41	94.85	97.94	91.75	83.51	88.66	91.75	88.66	91.03 ± 5.52
	fTCD	69.71	49.48	42.27	58.76	57.73	57.73	48.45	45.36	47.42	49.48	52.58 ± 7.79
	Fusion	97.92	96.88	95.83	94.79	96.88	95.83	88.54	95.83	95.83	90.62	94.9 ± 2.81
Baseline vs Right	EEG	98.96	90.62	91.67	81.25	95.83	97.92	83.33	89.58	94.79	83.33	90.73 ± 6.04
	fTCD	48.96	46.88	48.96	66.67	46.88	60.42	51.04	48.96	38.54	62.5	51.98 ± 8.13
	Fusion	97.89	94.74	91.58	92.63	96.84	96.84	89.47	97.89	95.79	91.58	94.53 ± 2.86
Right vs Left	EEG	99.05	77.14	83.81	96.19	94.29	92.38	83.81	85.71	98.1	94.29	90.48 ± 6.98
	fTCD	51.43	69.52	49.52	48.57	60.95	53.33	47.62	41.9	48.57	42.86	51.43 ± 7.89
	Fusion	99.05	85.71	89.52	98.1	100	99.05	94.29	97.14	100	100	96.29 ± 4.72

https://doi.org/10.1371/journal.pone.0311075.t001

Table 2. P-values showing accuracy significance of weighted probabilistic fusion compared to EEG only for the MI paradigm.

Comparison	Baseline vs Right MI	Baseline vs Left MI	Right vs Left MI
Fusion/EEG	0.0420	0.0098	0.0020

https://doi.org/10.1371/journal.pone.0311075.t002

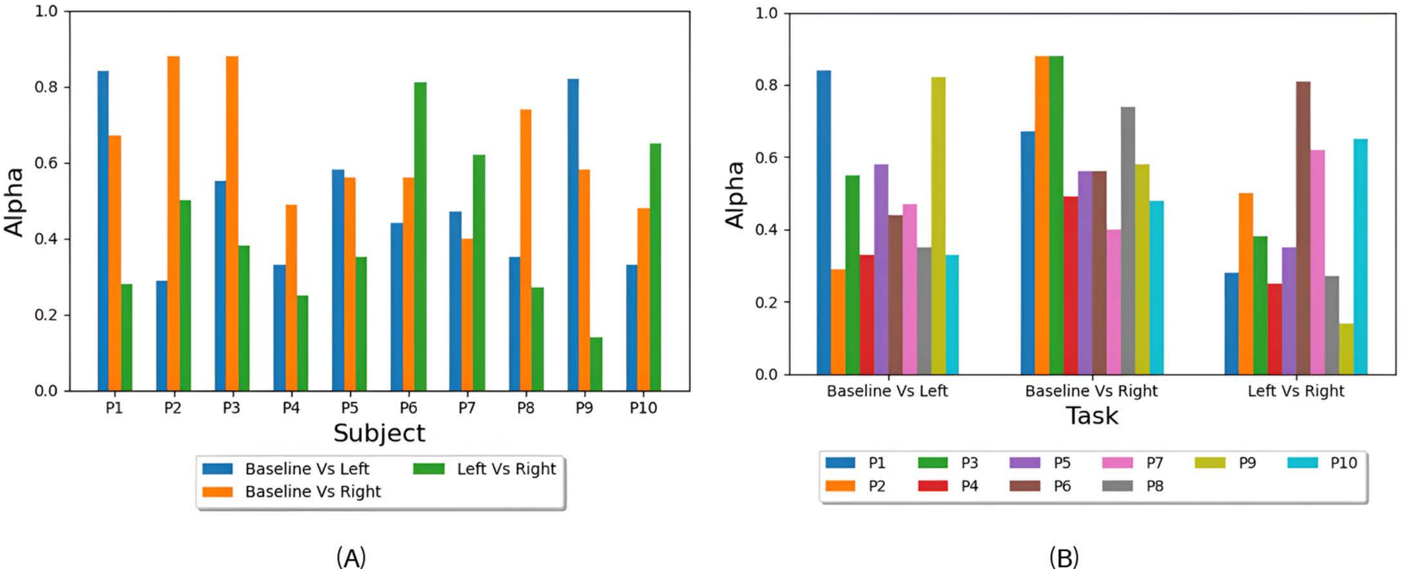

Fig 5. Optimal alpha values: (A) the values of alpha yielding the highest accuracy for each subject in Baseline Vs Left, Baseline Vs Right, and Left Vs Right tasks, and (B) average values of alpha yielding the highest accuracy for each task (Baseline Vs Left, Baseline Vs Right, and Left Vs Right).

https://doi.org/10.1371/journal.pone.0311075.g005

shows an average accuracy of 96.29% ± 4.72 for the weighted probabilistic fusion which outperforms the accuracies of 90.48% ± 6.98 and 51.43% ± 7.89 obtained using EEG only and fTCD only respectively. In comparison with EEG only, the weighted probabilistic fusion model shows significance with a p-value of 0.002 as shown in Table 2. When compared to feature vector concatenation, weighted probabilistic fusion demonstrated superior performance (see Supplementary S1 and S3 Tables). Additionally, the same analyses were conducted using an LDA classifier instead of SVM; however, SVM achieved higher accuracy (see Supplementary S1–S4 Tables).

The optimal alpha values that give the highest accuracy for each subject are reported in Fig 5A and the average of these values per task is reported in Fig 5B. These alpha values represent the contribution of each of the two modalities in decision making in the three classification problems. More specifically, alpha is the weighting factor for EEG modality and (1-alpha) is the fTCD weighting factor, so higher alpha values reflect higher EEG contribution in discriminating the tasks at the given problem. To test whether the contribution of EEG and fTCD modalities in decision making is task-dependent, one-sided Wilcoxon rank-sum test with a p-value of 0.05 was applied to the optimal alpha values of each subject within each task to check if their distribution have a median lower or higher than 0.5 where 0.5 represents equal EEG and fTCD contributions. No significance was observed for the three classification problems.

3.2. Flickering MR/WG Paradigm

The maximum accuracy achieved per subject using weighted probabilistic fusion and the corresponding EEG only accuracy and fTCD only accuracy are reported in Table 3 for MR versus baseline, WG versus baseline, and MR versus WG problems. Table 4 shows the calculated p-values using one-sided Wilcoxon signed rank test to compare the significance

Table 3. Maximum accuracy achieved for each subject using weighted fusion and the corresponding accuracies obtained using EEG only and fTCD only for flickering MR/WG paradigm.

	Sub_ID	1	2	3	4	5	6	7	8	9	10	11	Mean± STD
Baseline vs MR	EEG	92.71	96.88	88.54	88.54	91.67	89.58	91.67	97.92	98.96	95.83	90.62	92.99±3.62
	fTCD	41.67	44.79	55.21	59.38	63.54	39.58	48.96	44.79	59.38	41.67	51.04	50.0± 7.93
	Fusion	93.75	96.88	89.58	91.67	96.88	95.83	96.88	96.88	98.96	95.83	94.79	95.27± 2.58
Baseline vs WG	EEG	89.69	85.57	83.51	76.29	73.2	83.51	81.44	91.75	94.85	59.79	71.13	80.98± 9.77
	fTCD	58.76	55.67	63.92	50.52	71.13	50.52	42.27	39.18	57.73	42.27	43.3	52.3± 9.69
	Fusion	91.58	92.63	89.47	72.63	85.26	86.32	87.37	93.68	95.79	67.37	83.16	85.93± 8.42
MR vs WG	EEG	96.19	99.05	91.43	87.62	97.14	99.05	87.62	98.1	100	96.19	97.14	95.41± 4.27
	fTCD	49.52	49.52	45.71	49.52	44.76	57.14	66.67	44.76	56.19	41.9	53.33	50.82± 6.82
	Fusion	97.14	99.05	98.1	86.67	97.14	100	95.24	98.1	99.05	98.1	98.1	96.97± 3.48

https://doi.org/10.1371/journal.pone.0311075.t003

Table 4. P-values showing accuracy significance of weighted fusion compared to EEG only for flickering MR/WG paradigm.

Comparisons	Baseline vs MR	Baseline vs WG	MR vs WG
Fusion/EEG	0.0156	0.0049	0.0469

https://doi.org/10.1371/journal.pone.0311075.t004

of the weighted probabilistic fusion with EEG only in terms of accuracy. MR versus baseline problem achieved average accuracies of 95.27% ± 2.58 for the weighted probabilistic fusion model which is higher than 92.99% ± 3.62 for EEG only and 50% ± 7.93 for fTCD only (Table 3). Table 4 shows a p-value of 0.0156 describing the statistical significance of the increase in accuracy of the weighted probabilistic fusion in comparison with EEG only. Table 3 also shows the performance measures for WG versus baseline problem. In particular, weighted probabilistic fusion achieved average accuracy

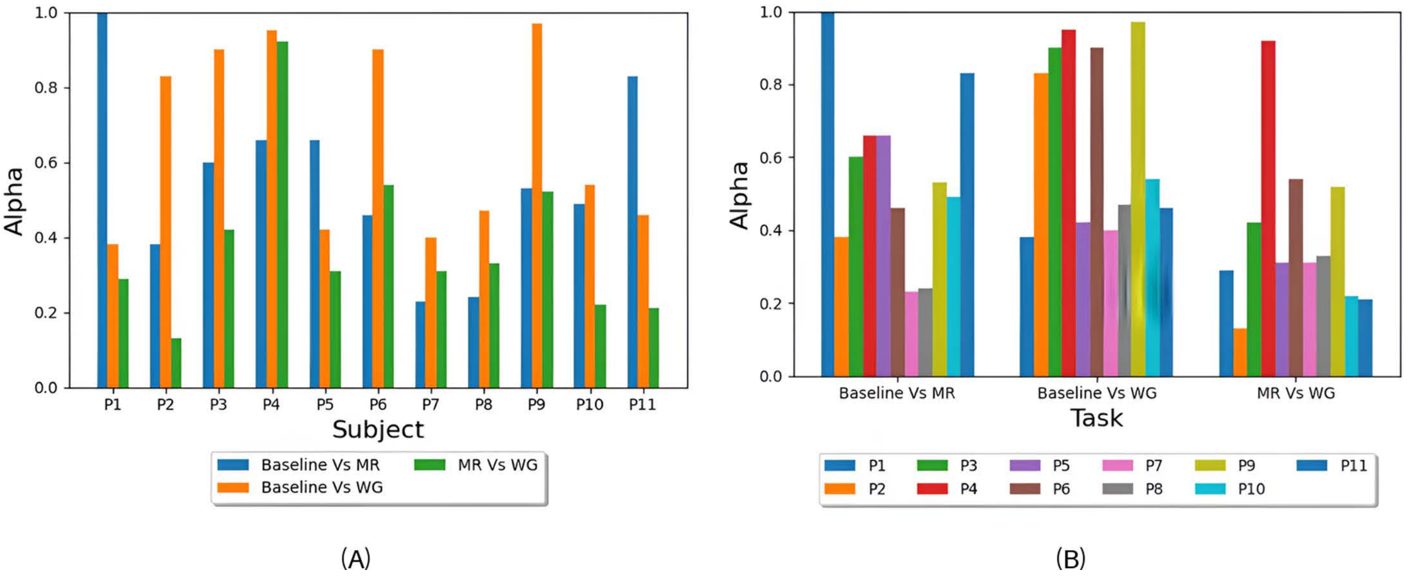

Fig 6. Optimal alpha values: (A) the values of alpha yielding the highest accuracy for each subject in Baseline Vs MR, Baseline Vs WG, and MR Vs WG tasks, and (B) average values of alpha yielding the highest accuracy for each task (Baseline Vs MR, Baseline Vs WG, and MR Vs WG).

https://doi.org/10.1371/journal.pone.0311075.g006

of 85.93% ± 8.42 compared to 80.98% ± 9.77 for EEG only and 52.3% ± 9.69 for fTCD only. Table 4 proves the significance of the weighted probabilistic fusion in terms of accuracy compared to EEG only with a p-value of 0.0049. As for MR versus WG problem, we obtained the highest average accuracy compared to MR/WG versus baseline problems as seen in Table 3. In particular, average accuracy of 96.97% ± 3.48 for the weighted probabilistic fusion was obtained which outperformed the average accuracy of 95.41% ± 4.27 and 50.82% ± 6.82 obtained using EEG only and fTCD only respectively. Table 4 shows a p-value of 0.0469 for the weighted fusion which indicates that it is statistically significant compared to EEG only. The weighted probabilistic fusion demonstrated superior performance when compared to feature vector concatenation (see Supplementary S5 and S7 Tables). Additionally, the same analyses were conducted using LDA instead of SVM; however, SVM yielded higher accuracy (see Supplementary S5–S8 Tables).

The optimal alpha values for each subject are reported in Fig 6A and the average of these values per task is reported in Fig 6B. Statical significance testing similar to the one performed in section 3.1 was applied to the alpha values of each task. Baseline versus MR and baseline versus WG tasks showed no significance while the alpha values of MR versus WG task was significantly lower than 0.5 with a p-value of 0.03.

4. Discussion

To enhance the performance of MI and MR/WG paradigms, we employed FBCSP to analyze EEG data of both paradigms and extracted time series features from the envelope of fTCD signals of both paradigms. Probabilistic Bayesian fusion was used to infer user intent based on EEG and fTCD input. Probabilistic fusion obtained statistically significant higher accuracies than EEG only by 3.87%, 3.80%, and 5.81% on average for baseline versus left MI, baseline versus right MI, and right MI versus left MI respectively (Table 1). Interestingly, FBCSP which is known as a successful feature extraction method for MI-based BCIs, yielded high accuracy when used to analyze EEG data of the MR/WG SSVEP paradigm. In fact, performance measures obtained with FBCSP outperform the feature extraction methods we used in previous studies for MR/WG paradigm [45,48]. Average accuracies of 95.27%, 85.93%, and 96.97% were obtained for MR versus baseline, WG versus baseline, and MR versus WG respectively using Bayesian fusion with average increases of 2.28%, 4.95%, and 1.56% compared to EEG only (Table 2). It can be noted that the average accuracy for the WG versus baseline problem is much lower than the average accuracies of MR versus baseline and WG versus baseline. The reason for such accuracy drop is unknown based on the available data, but requires further investigations. Although fTCD only performance accuracy was low for both paradigms, it was able to improve the multimodal performance when combined with EEG. Moreover, using the envelope fTCD signal instead of the raw signal has significantly sped up computations, especially in feature extraction, as the envelope signal has much lower dimensionality. This increase in computational efficiency made it possible to calculate several sets of features that were impossible to be calculated on the raw fTCD signal due to their computational complexity. By employing TSFEL as a comprehensive feature extraction approach, we were able to explore different characteristics of the envelope signal rather than limiting the analysis to a specific feature set. For example, while previous work [43] focused solely on statistical features derived from wavelet coefficients, we broadened our analysis by including statistical, spectral, and temporal features. In real-time BCI applications, testing time is critical, as it directly impacts system responsiveness and usability. The training time for the MI and MR/WG paradigms was found to be 200 seconds per subject, while the testing time for a single trial was 0.026 seconds. The low testing time of our approach makes it well-suited for real-time BCI applications, ensuring rapid feedback and an improved user experience.

The contribution of each modality to user intent inference was investigated per task, revealing that EEG had significantly lower contribution than fTCD in the WG versus MR task. No other tasks showed a clear advantage for either modality. This discrepancy between the MR/WG and MI paradigms may stem from the fact that the MI paradigm imposes the same cognitive demands for both EEG and fTCD, whereas the MR/WG paradigm involves distinct cognitive requirements for each modality. Specifically, in the MI paradigm, both EEG and fTCD capture changes driven by motor imagery. In contrast, in the MR/WG paradigm, EEG changes are primarily driven by the flickering of the MR/WG stimuli, whereas fTCD changes are

driven by the mental imagery/analytical processes required for performing the task. These differences in cognitive demands and task complexity between EEG and fTCD in the MR/WG paradigm may lead participants to perform better in aspects of the task that align more closely with the strengths of one modality over the other. While EEG showed a significantly lower contribution than fTCD in the MR versus WG task, no significant difference between EEG and fTCD contributions was observed in the MR/WG versus baseline tasks. We believe this discrepancy arises because the baseline cross was positioned very close to the flickering MR and WG stimuli, causing the flickering to capture participants' attention even when focusing on the baseline cross, thereby hindering EEG's ability to distinguish between the MR/WG and baseline.

The observed differences in EEG and fTCD contributions across tasks may reflect the distinct sensitivities of these modalities in detecting task-related neural or hemodynamic changes. Across subjects, these variations may be attributed to several factors. Individual differences in task performance may arise, as some participants may find the demands of one modality more intuitive or easier to engage with than the other. Additionally, the effects of fatigue can vary across individuals. For instance, one participant may experience early fatigue due to exposure to the flickering stimuli in the MR/WG paradigm, impairing their ability to engage in the mental imagery and analytical processes required for fTCD. In contrast, another participant may experience fatigue later in the task, leading to different effects on modality-specific contributions. Further variability may be introduced by differences in task execution strategies, mental states, and baseline neural/hemodynamic activity before task initiation. To fully understand these variations in modality-specific contributions, both across- and within-subject variability in EEG and fTCD contributions should be further investigated, with larger sample sizes being necessary to uncover the underlying mechanisms driving these contributions and their impact on task performance.

Table 5 shows a comparison between EEG-fTCD performance obtained using the proposed analysis approach and the performance we obtained in previous studies [47,48]. For task versus baseline problems in both paradigms, similar or higher accuracies were obtained with the current analysis pipeline compared to those we obtained previously. In particular, average accuracies of 95.27% and 85.93% were obtained for MR versus baseline and WG versus baseline problems respectively with the proposed analysis approach compared to 86.27% and 85.29% obtained previously. In addition, accuracies of 94.53% and 94.9% were achieved for left MI versus baseline and right MI versus baseline respectively compared to 93.71% and 93.85% obtained in previous studies. However, the accuracy of the proposed approach dropped by 1.2% and 3.7% for MR versus WG and left MI versus right MI respectively compared to our previous results. Despite the drop in task versus task accuracy, we believe the current analysis approach is more successful than the approaches we introduced earlier especially for MR/WG paradigm as it led to a significant 9% increase in performance accuracy for the MR versus baseline problem.

Table 5. Comparison between the EEG-fTCD BCI performance with proposed analysis approach and the approaches introduced previously.

Method	Modality	Activity	Accuracy	Task-Baseline/rest length (s)
[47] Khalaf et al.,2019 (Left/baseline)	EEG+fTCD	Motor Imagery	93.71%	10 – NA
[47] Khalaf et al.,2019 (Right/baseline)	EEG+fTCD	Motor Imagery	93.85%	10 – NA
[47] Khalaf et al.,2019 (Left/Right)	EEG+fTCD	Motor Imagery	100.00%	10 – NA
[48] Khalaf et al.,2019 (MR/baseline)	EEG+fTCD	Flickering MR/WG	86.27%	10 – NA
[48] Khalaf et al.,2019 (WG/baseline)	EEG+fTCD	Flickering MR/WG	85.29%	10 – NA
[48] Khalaf et al.,2019 (MR/WG)	EEG+fTCD	Flickering MR/WG	98.18%	10 – NA
Proposed Method (Left/baseline)	EEG+fTCD	Motor Imagery	94.53%	10 – NA
Proposed Method (Right/baseline)	EEG+fTCD	Motor Imagery	94.9%	10 – NA
Proposed Method (Left/Right)	EEG+fTCD	Motor Imagery	96.29%	10 – NA
Proposed Method (MR/baseline)	EEG+fTCD	Flickering MR/WG	95.27%	10 – NA
Proposed Method (WG/baseline)	EEG+fTCD	Flickering MR/WG	85.93%	10 – NA
Proposed Method (MR/WG)	EEG+fTCD	Flickering MR/WG	96.97%	10 – NA

https://doi.org/10.1371/journal.pone.0311075.t005

As shown in Table 6, our system outperforms all multimodal EEG-fNIRS BCIs in terms of accuracy in all three binary selection tasks in the MI paradigm and in two out of three binary selection tasks in the MR/WG paradigm. In addition, our system has the shortest trial length compared to all the multimodal BCIs in comparison expect for the BCI by Buccino et al. [60], however, that BCI requires a rest period of 6 seconds in addition to 6 seconds trial length while our system does not require any rest periods between trials. In fact, the rest task in our system (when the user focuses on the fixation cross) is randomly selected by the vertical arrow in the visual paradigm and is considered a separate task resembling the situation when the subject does not want to issue any commands while in the other BCIs in comparison, each trial is followed by a rest period to stabilize the hemodynamic response before the next trial. Our study specifically aimed to

Table 6. Comparison between MI and flickering WG/MR EEG-fTCD multimodal BCIs with the proposed analysis approach and the state-of-the-art multimodal EEG-fNIRS in literature.

Study	Modality	Activity	EEG features	Second modality features	Classification and fusion	Accuracy	Task-Baseline/ rest length (s)
[63] Fazli et al., 2012	EEG + fNIRS	Motor Imagery	CSP	HbO + HbR timeseries features	LDA with meta-classifier	83.20%	5–6/0
[64] Blokland et al., 2014	EEG + fNIRS	Motor Imagery	Power spectral features	O2Hb + HHb timeseries features	SVM with Wavelet-based multi-resolution fusion	79.00%	15–0/30±3
[62] Yin et al., 2015	EEG + fNIRS	Motor Imagery	Power spectral features, instantaneous Phase, Amplitude and Frequency	HbO + HbR timeseries features,	Extreme learning machines with Joint mutual information	89.00%	10–0/21±1
[65] Koo et al. 2015	EEG + fNIRS	Motor Imagery	CSP	HbO timeseries features	Linear SVM	88.00%	15–0/21±1
[60] Buccino et al., 2016	EEG + fNIRS	Motor Execution	CSP	HbO + HbR timeseries features	LDA with concatenation	72.20%	6–6/0
[60] Buccino et al., 2016	EEG + fNIRS	Motor Execution	CSP	HbO + HbR timeseries features	LDA with concatenation	94.20%	6–6/0
[66] Shin et al., 2017	EEG + fNIRS	Mental Arithmetic	CSP	HbO + HbR timeseries features	LDA with metaclassifier	88.20%	10–0/16±1
[40] Shin et al., 2018	EEG + fNIRS	Mental Imagery & Mental Arithmetic	CSP	HbO + HbR timeseries features	LDA with metaclassifier	82.2 ± 10.2%	10–0/17±1
[61] Wang et al., 2021	EEG + fNIRS	Motor Imagery	Dynamic functional connectivity	Dynamic functional connectivity	LSTM with Fully Connected Layers	90.03%	15-0/15
Proposed Method (MR/baseline)	EEG + fTCD	Flickering MR/ WG	FBCSP	fTCD envelope features	SVM with Bayesian fusion	95.27%	10 – NA
Proposed Method (WG/baseline)	EEG + fTCD	Flickering MR/ WG	FBCSP	fTCD envelope features	SVM with Bayesian fusion	85.93%	10 – NA
Proposed Method (MR/WG)	EEG + fTCD	Flickering MR/ WG	FBCSP	fTCD envelope features	SVM with Bayesian fusion	96.97%	10 – NA
Proposed Method (Left/baseline)	EEG + fTCD	Motor Imagery	FBCSP	fTCD envelope features	SVM with Bayesian fusion	94.53%	10 – NA
Proposed Method (Right/baseline)	EEG + fTCD	Motor Imagery	FBCSP	fTCD envelope features	SVM with Bayesian fusion	94.9%	10 – NA
Proposed Method (Left/Right)	EEG + fTCD	Motor Imagery	FBCSP	fTCD envelope features	SVM with Bayesian fusion	96.29%	10 – NA

https://doi.org/10.1371/journal.pone.0311075.t006

investigate the feasibility of fTCD-based BCI without enforced rest periods to enhance speed and efficiency. Our previous study on fTCD-based BCIs [43] has incorporated rest periods and demonstrated significantly higher accuracies and information transfer rates compared to fNIRS-based BCIs. Given these promising results, we aimed to evaluate whether a continuous operation mode – without explicit rest periods – could maintain high accuracy while further improving the system's responsiveness. Future work may further explore the impact of explicitly reintroducing rest periods to assess potential trade-offs between speed, accuracy, and user adaptability.

Additionally, the study that achieved the highest EEG-fNIRS accuracy of 94.2% used a motor execution task [60] while our system does not require any muscular input, making it more suitable for individuals with severe motor disabilities. The 85.93% accuracy achieved in the WG versus baseline problem is lower than the accuracies obtained by several EEG-fNRIS systems [60–64]. However, these systems are either much slower due to rest periods or require muscular input. Notably, our results were achieved with a significantly simpler setup requiring only two ultrasound transducers for fTCD compared to the multiple fNIRS optodes, making it both simpler to set up and more portable than fNIRS. Overall, our findings indicate that EEG-fTCD BCIs can serve as a viable alternative to EEG-fNIRS systems, offering higher accuracy, faster response times, and suitability for motor-impaired users, making them a promising direction for future multimodal BCI development.

Although both fNIRS and fTCD measure cerebral blood dynamics, they capture different physiological signals–fNIRS monitors changes in oxygenated and deoxygenated hemoglobin, whereas fTCD measures cerebral blood flow velocity in major cerebral arteries. These differences in measurement principles may impact the sensitivity of each modality to different cognitive and motor tasks. Additionally, fTCD has been shown to be a faster alternative compared to fNIRS in BCI design [43]. Differences in performance may also arise from the nature of the tasks used in each study. The EEG-fNIRS studies in comparison primarily employed motor execution [cite refs], motor imagery, and mental arithmetic tasks. Within the motor imagery paradigms, specific tasks included imagining finger/thumb tapping [64], hand clenching at different speeds and forces [62], and hand grasping [65]. Beyond task-related differences, variations in feature extraction and classification methods could also contribute to performance discrepancies. EEG-fNIRS studies have commonly used CSP for EEG features, while basic time series features of blood oxygenation changes were employed for fNIRS analysis [40,60,63,65,66]. In contrast, our approach employs FBCSP for EEG feature extraction and a comprehensive set of features for fTCD analysis. Additionally, our study incorporates a probabilistic Bayesian fusion framework that allows unequal weighting of EEG and fTCD contributions during classification unlike the studies in comparison which rely mainly on feature vector concatenation or meta-classifiers [40,60,63,66]. These differences in feature extraction, classification, and information fusion techniques may significantly impact how well user intent can be inferred from the recorded signals, ultimately influencing overall system performance.

Our study has several limitations that should be addressed in future work. One limitation is the low performance accuracy of fTCD only. Additional fTCD envelope and raw fTCD signal features should be investigated to improve fTCD accuracy and enhance multimodal performance. Moreover, our approach to fTCD feature extraction is exploratory due to the absence of established fTCD feature sets in the literature. While we extracted a broad set of statistical, spectral, and temporal features for fTCD signals, future work should investigate the relative importance of different feature sets and identify the most relevant features for each paradigm to enhance classification performance and reduce computational complexity. As for EEG feature extraction, future studies should consider phase-based features, such as the instantaneous phase difference sequence, which has shown potential in EEG-based motor imagery classification. Another limitation is the lack of explanation of the accuracy drop in WG versus baseline problem compared to the other selection problems in the MR/WG paradigm, which requires a larger sample size for further investigation. The Bayesian fusion approach introduces an additional hyperparameter, as it requires optimizing the weight of each modality per subject to achieve optimal decision-making. While this adds complexity, it also provides flexibility by allowing the fusion process to adapt to individual differences in modality contributions. Additionally, the small dataset size limits our ability

to assess the relative contributions of EEG and fTCD across different tasks and paradigms. A larger sample size would allow for a more robust investigation of how each modality influences user intent inference. Furthermore, the current framework does not evaluate the relative importance of extracted features (e.g., statistical, temporal, and spectral features). Future studies should focus on identifying the most discriminative feature types to improve performance and reduce computational demands. While our offline analysis demonstrates promising results, the system still requires validation in an online setting, where the trained pipeline processes continuous EEG and fTCD data streams in real time. Our analysis approach did not include cross-subject analysis, meaning that model performance was evaluated on a subject-specific basis. However, reducing calibration time and improving model generalizability across subjects is a crucial next step. Future work should explore invariant representation learning and transfer learning techniques to identify shared neural activity patterns in the EEG-fTCD joint space. Leveraging these invariant features could enable cross-subject decoding models, reducing the need for extensive per-user calibration while enhancing the system's adaptability across different users and recording sessions.

5. Conclusion

In this paper, we propose a machine learning approach to improve the performance of multimodal EEG-fTCD BCIs. Specifically, we employed FBCSP and time series features to analyze EEG signals and the envelope of fTCD signals, respectively. To integrate information from EEG and fTCD, we applied a probabilistic fusion approach, which outperformed feature vector concatenation. Binary selection problems were investigated for both MI and flickering MR/WG paradigms. For the MR/WG paradigm, the multimodal system achieved average accuracies of 95.27% ± 2.58, 85.98% ± 8.42, and 96.97% ± 3.48 for baseline versus MR, baseline versus WG, and MR versus WG respectively. The MI paradigm achieved average accuracies of 94.53% ± 2.81, 94.9% ± 2.86, and 96.29% ± 4.72 for baseline versus left, baseline versus right, and left versus right, respectively. Compared to existing models applied to the same dataset, our findings demonstrate that the proposed approach achieves better performance in most classification tasks. Additionally, the multimodal EEG-fTCD BCI with the proposed analysis pipeline outperforms all EEG-fNRIS BCIs in comparison. The proposed EEG-fTCD system significantly advances multimodal BCIs, offering portability, cost-effectiveness, ease of setup, and superior performance compared to other multi-modal systems like EEG-fMRI, EEG-MEG, and EEG-fNIRS. These advantages make it a promising and user-friendly solution for individuals with severe motor or speech disabilities.

Supporting information

S1 Table. Maximum accuracy achieved for each subject using SVM and the corresponding accuracies obtained using Concatenation and fusion for MI paradigm.
(DOCX)

S2 Table. Maximum accuracy achieved for each subject using LDA and the corresponding accuracies obtained using Concatenation and fusion for MI paradigm.
(DOCX)

S3 Table. P-values showing accuracy significance of fusion compared to Concatenation for the MI paradigm with SVM.
(DOCX)

S4 Table. P-values showing accuracy significance of fusion compared to Concatenation for the MI paradigm with LDA.
(DOCX)

S5 Table. Maximum accuracy achieved for each subject using SVM and the corresponding accuracies obtained using Concatenation and fusion for MR/WG paradigm.
(DOCX)

S6 Table. Maximum accuracy achieved for each subject using LDA and the corresponding accuracies obtained using Concatenation and fusion for MR/WG paradigm.
(DOCX)

S7 Table. P-values showing accuracy significance of fusion compared to Concatenation for the MR/WG paradigm with SVM.
(DOCX)

S8 Table. P-values showing accuracy significance of fusion compared to Concatenation for the MR/WG paradigm with LDA.
(DOCX)

Author contributions

Conceptualization: Aya Khalaf.

Data curation: Alaa-Allah Essam, Ammar Ibrahim, Aya Khalaf, Ashar Seif Al-Nasr, Mariam El-Saqa, Sohila Mohamed.

Formal analysis: Alaa-Allah Essam, Ammar Ibrahim, Ashar Seif Al-Nasr, Mariam El-Saqa, Sohila Mohamed.

Investigation: Murat Akcakaya, Aya Khalaf.

Methodology: Murat Akcakaya, Alaa-Allah Essam, Ammar Ibrahim, Mariam El-Saqa, Sohila Mohamed, Ayman Anwar, Ayman Eldeib, Aya Khalaf.

Project administration: Aya Khalaf.

Resources: Murat Akcakaya.

Software: Alaa-Allah Essam, Ammar Ibrahim, Ashar Seif Al-Nasr, Mariam El-Saqa, Sohila Mohamed, Ayman Anwar.

Supervision: Ashar Seif Al-Nasr, Aya Khalaf.

Validation: Alaa-Allah Essam, Ammar Ibrahim, Ashar Seif Al-Nasr, Mariam El-Saqa, Sohila Mohamed.

Visualization: Alaa-Allah Essam, Ammar Ibrahim, Ashar Seif Al-Nasr, Mariam El-Saqa, Sohila Mohamed.

Writing – original draft: Alaa-Allah Essam, Ammar Ibrahim, Ashar Seif Al-Nasr, Mariam El-Saqa.

Writing – review & editing: Ayman Anwar, Ayman Eldeib, Murat Akcakaya, Aya Khalaf.

References

1. Nicolas-Alonso LF, Gomez-Gil J. Brain computer interfaces, a review. Sensors (Basel). 2012;12(2):1211–79. https://doi.org/10.3390/s120201211 PMID: 22438708
2. Khan MU, Hasan MAH. Hybrid EEG-fNIRS BCI Fusion Using Multi-Resolution Singular Value Decomposition (MSVD). Front Hum Neurosci. 2020 Dec 8;14.
3. Lazarou I, Nikolopoulos S, Petrantonakis PC, Kompatsiaris I, Tsolaki M. EEG-Based Brain–Computer Interfaces for Communication and Rehabilitation of People with Motor Impairment: A Novel Approach of the 21st Century. Front Hum Neurosci. 2018 Jan 31;12.
4. Duan X, Xie S, Xie X, Meng Y, Xu Z. Quadcopter Flight Control Using a Non-invasive Multi-Modal Brain Computer Interface. Front Neurorobot. 2019 May 31;13.
5. Yanagisawa T, Fukuma R, Seymour B, Hosomi K, Kishima H, Shimizu T, et al. Using a BCI Prosthetic Hand to Control Phantom Limb Pain. 2019:43–52.
6. Khan RA, Naseer N, Qureshi NK, Noori FM, Nazeer H, Khan MU. fNIRS-based Neurorobotic Interface for gait rehabilitation. J Neuroeng Rehabil. 2018 Dec 5;15(1):7. https://doi.org/10.1186/s12984-018-0346-2 PMID: 29402310

7. Coogan CG, He B. Brain-computer interface control in a virtual reality environment and applications for the internet of things. IEEE Access. 2018;6:10840–9. https://doi.org/10.1109/ACCESS.2018.2809453 PMID: 30271700
8. Ahn M, Lee M, Choi J, Jun S. A review of brain-computer interface games and an opinion survey from researchers, developers and users. Sensors. 2014 Aug 11;14(8):14601–33. https://doi.org/10.3390/s140814601 PMID: 25116904
9. Waldert S. Invasive vs. Non-Invasive Neuronal Signals for Brain-Machine Interfaces: Will One Prevail?. Front Neurosci. 2016 Jun 27;10
10. Kwon J, Shin J, Im C-H. Toward a compact hybrid brain-computer interface (BCI): Performance evaluation of multi-class hybrid EEG-fNIRS BCIs with limited number of channels. PLoS One. 2020;15(3):e0230491. https://doi.org/10.1371/journal.pone.0230491 PMID: 32187208
11. Lotte F, Congedo M, Lécuyer A, Lamarche F, Arnaldi B. A review of classification algorithms for EEG-based brain–computer interfaces. J Neural Eng. 2007 Jun 1;4(2):R1–13.
12. Min B-K, Marzelli MJ, Yoo S-S. Neuroimaging-based approaches in the brain-computer interface. Trends Biotechnol. 2010 Nov;28(11):552–60. https://doi.org/10.1016/j.tibtech.2010.08.002 PMID: 20810180
13. Naseer N, Hong KS. fNIRS-based brain-computer interfaces: a review. Front Hum Neurosci. 2015 Jan 28;9.
14. Sokunbi MO, Gradin VB, Waiter GD, Cameron GG, Ahearn TS, Murray AD, et al. Nonlinear complexity analysis of brain fMRI signals in schizophrenia. PLoS One. 2014 May 13;9(5):e95146. https://doi.org/10.1371/journal.pone.0095146 PMID: 24824731
15. Lal SKL, Craig A, Boord P, Kirkup L, Nguyen H. Development of an algorithm for an EEG-based driver fatigue countermeasure. J Safety Res. 2003;34(3):321–8. https://doi.org/10.1016/s0022-4375(03)00027-6 PMID: 12963079
16. Aydemir O, Kayikcioglu T. Decision tree structure based classification of EEG signals recorded during two dimensional cursor movement imagery. J Neurosci Methods. 2014 May;229:68–75. https://doi.org/10.1016/j.jneumeth.2014.04.007 PMID: 24751647
17. Hasan MAH, Khan MU, Mishra D. A Computationally Efficient Method for Hybrid EEG-fNIRS BCI Based on the Pearson Correlation. Biomed Res Int. 2020 Aug 19; 2020:1–13.
18. Brandl S, Hohne J, Muller KR, Samek W. Bringing BCI into everyday life: Motor imagery in a pseudo realistic environment. In: 2015 7th International IEEE/EMBS Conference on Neural Engineering (NER). IEEE; 2015. 224–7.
19. Xu M. Han J, Wang Y, Jung T-P, Ming D. Implementing Over 100 Command Codes for a High-Speed Hybrid Brain-Computer Interface Using Concurrent P300 and SSVEP Features," in IEEE Transactions on Biomedical Engineering, Nov. 2020;67(11):3073–3082, https://doi.org/10.1109/TBME.2020.2975614
20. Zhang Y, Song J, Zhou S, Hu X, Zhang R. A novel hybrid P300-SSVEP BCI speller with frequency coding to improve spelling accuracy and speed. Frontiers in Neuroscience. 2023.
21. Jiang Y, Li G, Chen Y. Recent advances in P300-based BCI spellers: Strategies for accuracy and usability improvement. Frontiers in Human Neuroscience. 2023
22. Jin J, Wang Z, Xu R, Liu C, Wang X, Cichocki A. Robust Similarity Measurement Based on a Novel Time Filter for SSVEPs Detection. IEEE Trans Neural Netw Learn Syst. 2023;34(8):4096–4105. https://doi.org/10.1109/TNNLS.2021.3118468 PMID: 34648459
23. Zhang D, Liu Y, Hou Y, Chen X, Wang Y. A high-speed SSVEP-based BCI using dry EEG electrodes. Scientific Reports. 2018; 8:14708.
24. Devlaminck D, Wyns B, Grosse-Wentrup M, Otte G, Santens P. Multisubject learning for common spatial patterns in motor-imagery BCI. Comput Intell Neurosci. 2011;2011:1–9. PMID: 22007194
25. Ang KK, Chin ZY, Zhang H, Guan C. Filter Bank Common Spatial Pattern (FBCSP) in Brain-Computer Interface. In: 2008 IEEE International Joint Conference on Neural Networks (IEEE World Congress on Computational Intelligence). IEEE; 2008;2390–7.
26. Jin J, Xiao R, Daly I, Miao Y, Wang X, Cichocki A. Internal Feature Selection Method of CSP Based on L1-Norm and Dempster-Shafer Theory. IEEE Trans Neural Netw Learn Syst. 2021;32(11):4814–25. https://doi.org/10.1109/TNNLS.2020.3015505 PMID: 32833646
27. Sharma R, Kim M, Gupta A. Motor imagery classification in brain-machine interface with machine learning algorithms: Classical approach to multilayer perceptron model. Biomedical Signal Processing and Control, 2022;71:103101. https://doi.org/10.1016/j.bspc.2021.103101
28. Turco A, Zappasodi F, Tombini M, Pellegrino G, Mattia D. Motor-Imagery EEG Signals Classification using SVM, MLP, and LDA Classifiers. Turkish Journal of Computer and Mathematics Education (TURCOMAT). 2021;12(10):2393–2405.
29. González-Moreno A., Panetsos F., Maestú F. Evaluating the Efficacy of Motor Imagery Classifiers: Linear Discriminant Analysis and a Multi-Layer Perceptron Neural Network. Journal of Human and Social Sciences Research, 2022;5(1):101–118.
30. Liang Z, Wang P, He J, Lan W, Leng Y, Wang R, et al. FDCN-C: A deep learning model based on frequency enhancement, deformable convolution network, and crop module for electroencephalography motor imagery classification. PLOS ONE. 2024;19(11):1–23.
31. Roy A. M. An efficient multi-scale CNN model with intrinsic feature integration for motor imagery EEG subject classification in brain-machine interfaces. Biomedical Signal Processing and Control. 2022;74:103496. https://doi.org/10.1016/j.bspc.2022.103496
32. Roy Y, Mazumder O, Dey N. Adaptive transfer learning-based multiscale feature fused deep convolutional neural network for EEG MI multiclassification in brain–computer interface. Eng Appl Artif Intell. 2022;112:105347. https://doi.org/10.1016/j.engappai.2022.105347
33. Zhang Y. Multiscale Spatial-Temporal Feature Fusion Neural Network for Motor Imagery Brain-Computer Interfaces IEEE J Biomed Health Inform. 2024 https://doi.org/10.1109/JBHI.2024

34. Wang Y, Jin J, Xu R, Daly I, Zhao X, Cichocki A. MOCNN: A Multiscale Deep Convolutional Neural Network for ERP-Based Brain-Computer Interfaces. IEEE Trans Cybern. 2024;54(9):5565–76. https://doi.org/10.1109/TCYB.2024.3390805 PMID: 38713574
35. Hong KS, Khan MJ. Hybrid Brain–Computer Interface Techniques for Improved Classification Accuracy and Increased Number of Commands: A Review. Front Neurorobot. 2017 Jul 24;11.
36. Mano M, Lécuyer A, Bannier E, Perronnet L, Noorzadeh S, Barillot C. How to Build a Hybrid Neurofeedback Platform Combining EEG and fMRI. Front Neurosci. 2017 Mar 21;11.
37. Allison B, Wolpaw E, Wolpaw J. Brain–computer interface systems: progress and prospects. Expert Rev Med Devices. 2007 Jul 9;4(4):463–74.
38. Buccino AP, Keles HO, Omurtag A. Hybrid EEG-fNIRS Asynchronous Brain-Computer Interface for Multiple Motor Tasks. PLoS One. 2016 Jan 5; 11(1):e0146610.
39. Khan MJ, Hong KS. Hybrid EEG–fNIRS-Based Eight-Command Decoding for BCI: Application to Quadcopter Control. Front Neurorobot. 2017 Feb 17;11.
40. Shin J, Kwon J, Im CH. A Ternary Hybrid EEG-NIRS Brain-Computer Interface for the Classification of Brain Activation Patterns during Mental Arithmetic, Motor Imagery, and Idle State. Front Neuroinform. 2018 Feb 23;12.
41. Zephaniah P, Kim JG. Recent functional near infrared spectroscopy based brain computer interface systems: Developments, applications and challenges. Biomed Eng Lett. 2014 Sep 18;4(3):223–30
42. Naseer N, Hong K-S. fNIRS-based brain-computer interfaces: a review. Front Hum Neurosci. 2015;9:3. https://doi.org/10.3389/fnhum.2015.00003 PMID: 25674060
43. Khalaf A, Sybeldon M, Sejdic E, Akcakaya M. A brain-computer interface based on functional transcranial doppler ultrasound using wavelet transform and support vector machines. J Neurosci Methods. 2018 Jan;293:174–82. https://doi.org/10.1016/j.jneumeth.2017.10.003 PMID: 29017899
44. Alexandrov A, Sloan MA, Wong LKS, Douville C, Razumovsky AY, Koroshetz WJ, et al. Practice standards for transcranial Doppler ultrasound: part I--test performance. J Neuroimaging [Internet]. 2007 Jan [cited 2022 Aug 24]; 17(1):11–8. Available from: https://pubmed.ncbi.nlm.nih.gov/17238867/
45. Khalaf A, Sejdic E, Akcakaya M. Towards optimal visual presentation design for hybrid EEG-fTCD brain-computer interfaces. J Neural Eng. 2018 Oct 1; 15(5):056019. https://doi.org/10.1088/1741-2552/aad46f PMID: 30021931
46. Khalaf A, Sejdic E, Akcakaya M. A novel motor imagery hybrid brain computer interface using EEG and functional transcranial Doppler ultrasound. J Neurosci Methods. 2019;313:44–53. https://doi.org/10.1016/j.jneumeth.2018.11.017 PMID: 30590086
47. Khalaf A, Sejdic E, Akcakaya M. Common spatial pattern and wavelet decomposition for motor imagery EEG-fTCD brain-computer interface. J Neurosci Methods. 2019 May 15; 320:98–106.
48. Khalaf A, Sejdic E, Akcakaya M. EEG-fTCD hybrid brain-computer interface using template matching and wavelet decomposition. J Neural Eng. 2019;16(3):036014. https://doi.org/10.1088/1741-2552/ab0b7f PMID: 30818297
49. Khalaf A, Sejdic E, Murat A. Three-Class EEG-fTCD Brain-Computer Interfaces. 2020 Aug 29 [cited 2022 Aug 24]; Available from:/articles/preprint/Three-Class_EEG-fTCD_Brain-Computer_Interfaces/12867260/1
50. Aggarwal S, Chugh N. Signal processing techniques for motor imagery brain computer interface: A review. Array. 2019;1–2:100003. https://doi.org/10.1016/j.array.2019.100003
51. Blankertz B, Tomioka R, Lemm S, Kawanabe M, Muller K. Optimizing Spatial filters for Robust EEG Single-Trial Analysis. IEEE Signal Process Mag. 2008;25(1):41–56. https://doi.org/10.1109/msp.2008.4408441
52. Novi Q, Guan C, Dat TH, Xue P. Sub-band Common Spatial Pattern (SBCSP) for Brain-Computer Interface. In: 2007 3rd International IEEE/EMBS Conference on Neural Engineering. IEEE; 2007:204–7.
53. Ang KK, Chin ZY, Wang C, Guan C, Zhang H. Filter Bank Common Spatial Pattern Algorithm on BCI Competition IV Datasets 2a and 2b. Front Neurosci. 2012;6.
54. Vansteensel MJ, Jarosiewicz B. Brain-computer interfaces for communication. 2020:67–85.
55. Ha KW, Jeong JW. Motor Imagery EEG Classification Using Capsule Networks. Sensors. 2019 Jun 27; 19(13):2854.
56. Ai Q, Chen A, Chen K, Liu Q, Zhou T, Xin S, et al. Feature extraction of four-class motor imagery EEG signals based on functional brain network. J Neural Eng. 2019 Apr 1;16(2):026032. https://doi.org/10.1088/1741-2552/ab0328 PMID: 30699389
57. Tan P, Wang X, Wang Y. Dimensionality reduction in evolutionary algorithms-based feature selection for motor imagery brain-computer interface. Swarm Evol Comput. 2020 Feb;52:100597. https://doi.org/10.1016/j.swevo.2019.100597
58. Sejdić E, Kalika D, Czarnek N. An analysis of resting-state functional transcranial Doppler recordings from middle cerebral arteries. PLoS One. 2013 Feb 6;8(2):e55405. https://doi.org/10.1371/journal.pone.0055405 PMID: 23405146
59. Rey D, Neuhäuser M. Wilcoxon-Signed-Rank Test. In: International Encyclopedia of Statistical Science. Berlin, Heidelberg: Springer Berlin Heidelberg; 2011:1658–9.
60. Buccino AP, Keles HO, Omurtag A. Hybrid EEG-fNIRS Asynchronous Brain-Computer Interface for Multiple Motor Tasks. PLoS One. 2016 Jan 5;11(1):e0146610.

61. Wang P, He J, Lan W, Yang H, Leng Y, Wang R, et al. A Hybrid EEG-fNIRS Brain-Computer Interface Based on Dynamic Functional Connectivity and Long Short-Term Memory. In: 2021 IEEE 5th Advanced Information Technology, Electronic and Automation Control Conference (IAEAC). IEEE; 2021:2214–9.
62. Yin X, Xu B, Jiang C, Fu Y, Wang Z, Li H, et al. A hybrid BCI based on EEG and fNIRS signals improves the performance of decoding motor imagery of both force and speed of hand clenching. J Neural Eng. 2015;12(3):036004. https://doi.org/10.1088/1741-2560/12/3/036004 PMID: 25834118
63. Fazli S, Mehnert J, Steinbrink J, Curio G, Villringer A, Müller K-R, et al. Enhanced performance by a hybrid NIRS-EEG brain computer interface. Neuroimage. 2012;59(1):519–29. https://doi.org/10.1016/j.neuroimage.2011.07.084 PMID: 21840399
64. Blokland Y, Spyrou L, Thijssen D, Eijsvogels T, Colier W, Floor-Westerdijk M, et al. Combined EEG-fNIRS Decoding of Motor Attempt and Imagery for Brain Switch Control: An Offline Study in Patients With Tetraplegia. IEEE Transactions on Neural Systems and Rehabilitation Engineering. 2014 Mar;22(2):222–9.
65. Koo B, Lee H-G, Nam Y, Kang H, Koh CS, Shin H-C, et al. A hybrid NIRS-EEG system for self-paced brain computer interface with online motor imagery. J Neurosci Methods. 2015;244:26–32. https://doi.org/10.1016/j.jneumeth.2014.04.016 PMID: 24797225
66. Shin J, Müller K-R, Schmitz CH, Kim D-W, Hwang H-J. Evaluation of a Compact Hybrid Brain-Computer Interface System. Biomed Res Int. 2017;2017:1–11. https://doi.org/10.1155/2017/6820482 PMID: 28373984
